# Primary open angle glaucoma in northern Nigeria: stage at presentation and acceptance of treatment

**DOI:** 10.1186/s12886-015-0097-9

**Published:** 2015-08-22

**Authors:** Mohammed M. Abdull, Clare C. Gilbert, Jennifer Evans

**Affiliations:** Ophthalmology Department, Abubakar Tafawa Balewa University Teaching Hospital, Bauchi, Bauchi State Nigeria; International Centre for Eye Health, Department of Clinical Research, London School of Hygiene & Tropical Medicine, London, UK

## Abstract

**Background:**

To determine the stage of primary open angle glaucoma at presentation at a tertiary eye unit, to assess patient’s knowledge of glaucoma and acceptance and subsequent adherence to treatment.

**Method:**

Information collected prospectively on new glaucoma patients aged 30 or more years included distance from residence and what they knew about glaucoma and its treatment. Treatment offered took account of disease severity and socioeconomic factors. Reasons for not accepting surgery were recorded. At follow up intraocular pressure (IOP) was measured and adherence to medication assessed verbally. Four categories of severity were defined based on visual acuity and visual fields defects in the worse eye.

**Results:**

131 patients were recruited (mean age 52.8 years; 62 % male). Most attended because of symptoms (70 %). Mean IOP in affected eyes was 31.9+/-SD 12.4 and mean vertical cup:disc ratio was 0.8. 99 eyes (47 %) had a visual acuity of light perception or worse. Risk factors for advanced/end-stage disease were age >50 years, living >10 km from the hospital, some awareness of glaucoma, not being literate, being unemployed and presenting with symptoms. In multivariable analysis older age and poor knowledge of glaucoma remained independent risk factors. 75 were offered trabeculectomy: five agreed but only one underwent surgery. Reasons for rejecting surgery were fear (37 %), preferred medical treatment (27 %) and cost (15 %). 32/85 (24 %) participants started on topical medication attended follow up. 72 % reported excellent compliance but only 56 % of glaucomatous eyes had IOPs less than 21mmHg.

**Conclusions:**

To prevent glaucoma blindness strategies are required which promote earlier detection, with counselling to promote acceptance of and adherence to treatment.

## Background

Glaucoma causes irreversible blindness in 4.6-6.7 million people worldwide [1]. In 2010 there were estimated to be 60.5 million people with glaucoma, which will reach 76.0 million by 2020 and 111.8 million by 2040. Primary open angle glaucoma (POAG) is the commonest form in Africa with the highest prevalence of any region (4.20 %; 95 % CI, 2.08-7.35) [2, 3]. The glaucoma-specific blindness prevalence in adults is estimated to be eight times higher in the two World Health Organization (WHO) African sub-regions than the Western Pacific region, which has the lowest prevalence [4]. In Nigeria, the prevalence of glaucoma is 5.02 % in adults 40 years and above (Kyari F, Entekume G, Rabiu M, Spry P et al. A population-based survey for the prevalence and types of glaucoma in Nigeria. The Nigeria national blindness and visual impairment survey. Submitted).

In many African patients, knowledge of the disease is poor as are acceptance of surgery and adherence to topical medication [5, 6]. Reasons for poor adherence include fear, lack of understanding that sight cannot be restored, and poverty. Community and family factors also contribute, as individuals’ decisions about expenditure on health impact on family resources leading to considerable delay in accessing treatment, if at all [7, 8]. This means that patients often present blind and at a relatively young age, as has been reported from several countries in Africa (Table 1).

**Table 1:** Visual acuity in the better eye among patients presenting to eye units with glaucoma in African countries

Country	Year	N	Age (mean years)	% Blind	Other findings
Northern Ghana [7]	1990	397	ND	52 % blind (VAc)	
Dar es Salam, Tanzania [38]	2005	298	57	29 % blind (VAc)	CDR ≥0.8: 70 %
Ethiopia [39]	2006	1,586	52	41 % blind (all glaucomas)	
Benin, Nigeria [40]	2006	154	53	25 % blind (VAc)	
				56 % blind (VFAc) 7	
Nigeria, Kano [41]	2007	71	18-75s	21 % blind (VAc)	Mean CDR 0.9;
					CDR ≥0.9: 30 %
Yaoundé, Cameroon [42]	2008	184	62	34 % blind (VAc)	
Dar Es Salam, Tanzania [6]	2009	163	67	Operated eyes: 47 % blind;	Pre-operative
				93 % VA <6/60	CDR 0.8: 85 %
Upper East Region, Ghana [43]	2010	446	34	No data	CDR > 0.8: 70 %
					CDR = 1.0: 54.9 %
Nigeria: This study	2010	131	53	35 % blind (Vac)	Mean CDR: 0.8
					CDR = 1.0: 44 %

*CDR* = cup:disc ratio; *VAc* = visual acuity criterion - presenting acuity <3/60 in the better eye; *VFAc* = visual field analysis criterion - central visual field of <10 degrees; *PL* = light perception; *NPL* = no light perception

The purpose of this study was to determine the stage of POAG at presentation at a tertiary eye unit in Nigeria, to assess patient’s knowledge of the disease and their acceptance and subsequent adherence to treatment, and to explore associations between stage at presentation and mode of presentation and socio-demographic variables.

The study was undertaken in Abubakar Tafawa Balewa University Teaching Hospital, (ATBUTH) Bauchi northeast Nigeria. The catchment area (i.e. within 250 km) is arid, with a population of approximately 4.3 million. The majority are Hausa speaking subsistence farmers, and education levels amongst adults are low (e.g. 65.7 % literacy in any language; 26.6 % in English) [9]. Life expectancy is approximately 48 years [10]. Poverty levels are high and infrastructure in terms of roads, public transport and electricity supplies is poor.

## Methods

For inclusion in this study, patients had to have POAG, have lived in the catchment area for at least 6 months, be aged ≥30 years, be a new glaucoma patient to the hospital (i.e. including referrals), be English or Hausa speaking and willing to participate. Those with additional causes of visual loss were excluded.

Potential participants were identified by examination based on symptoms at presentation, or by routine disc examination of all patients aged ≥30 years. For the latter, undilated disc examination was performed by direct ophthalmoscopy by one of two senior ophthalmic nurses or optometrists trained in optic disc examination. Anyone with a vertical cup:disc ratio (VCDR) of ≥0.6 was referred to the ophthalmologist (MA) for detailed examination. This VCDR was selected as data from the Nigeria national survey of blindness where a VCDR of 0.7 was identified as the cut-off for defining level 1 evidence of structural disc damage due to glaucoma [11]. Examination included presenting and unaided distance visual acuity (VA) measured in each eye by an ophthalmic nurse using a Snellen E chart. Best corrected VA was assessed using readings from an autorefractor (Takagi, Japan); the swinging flashlight test was performed for relative afferent pupillary defects, and anterior segments were examined at the slit-lamp (CSO, Italy). Other assessments included Von Herrick’s peripheral anterior chamber depth, IOP measurement (Goldman applanation tonometry), gonioscopy where possible, and optic disc examination using a 60D lens to assess VCDR, cup disc asymmetry and the presence of splinter haemorrhages or a notch. If slit lamp disc assessment was not possible, discs were examined by dilated binocular indirect ophthalmoscopy. Automated perimetry was performed using the screening program of the Oculus Twinfield visual field analyser (VFA) followed by threshold testing if defects consistent with glaucoma were detected. The diagnosis of POAG was made using internationally accepted guidelines [12] based on VCDR, visual field defect (VFD), IOP and an open angle on gonioscopy. Those confirmed with glaucoma who were eligible were recruited after taking written informed consent.

Glaucoma was graded by eye, and then by person using the worst affected eye. The following definitions were used: end stage: VA hand movement or worse and VCDR of 1.0; advanced: central VF of <10^0^ or VA < 3/60 in the presence of VCDR >0.8; moderate: central VF 10-20^0^ with VCDR >0.7 with any level of VA; mild: any other glaucomatous VFD and a VCDR >0.7. A VCDR of >0.7 was used as this defined the 95^th^ percentile in the Nigeria national survey normative dataset [11]. In our study advanced and end stage disease both fulfil WHO VA and VF categories of blindness.

The following information was obtained by interview: age, sex, literacy, occupation, distance from residence to the hospital, family history of visual loss or glaucoma, whether they knew they had glaucoma and if so, when and where it was diagnosed and previous treatment. Participants were asked what they knew about glaucoma and its treatment, and responses were graded using a four-point scale ranging from poor (i.e. they had never heard of glaucoma) through to excellent (i.e. they knew it is associated with high IOP or causes optic nerve damage or VFD). Knowledge of treatment was categorized as poor if they knew nothing about it through to excellent if they knew that glaucoma is treatable and could name a treatment.

Participants were offered treatment after explaining the condition and treatment options, taking account of the stage of disease, other clinical parameters and socioeconomic factors. Whether they agreed to the treatment recommended was recorded as well as reasons for not agreeing. Participants agreeing to surgery were started on topical treatment and given a date within two months to attend for surgery. Those recommended topical treatment were asked to re-attend in one month for IOP measurement and to assess adherence to medication. Adherence was assessed verbally and categorised as excellent if they had only missed very few doses, average, or very poor if they took only few to no doses.

Ethical approval was obtained from the ethical and research committees at London School of Hygiene & Tropical Medicine and ATBUTH. This study adhered to the tenets of the declaration of Helsinki.

### Data analysis

Data were entered and analysed using Stata 11.2 StataCorp LP. The eye with the most advanced glaucoma was used in the analysis for comparison with other studies. Data were analysed using two levels of severity: end-stage plus advanced, and mild plus moderate glaucoma. Age was categorized as ≥50 years and <50 years. Univariate and multivariate analyses were undertaken to assess associations between stage of disease and mode of presentation, age, sex, ethnicity, distance to place of residence, literacy, occupation and family history of glaucoma.

## Results

During the study (May-Sept 2010) 6,291 patients attended the outpatient department, 1,692 of whom were aged ≥30 years. 209 individuals were examined by the ophthalmologist based on a VCDR ≥0.6 in one or both eyes, 131 of whom were diagnosed with POAG i.e. 7.9 % (131/1692) of adult clinic attendants. The mean age of the 131 participants was 52.8 years (range 30-87 years), 62 % were male and 111 (90 %) had bilateral glaucoma (Table 2).

**Table 2:** Characteristics participants with POAG, by stage of glaucoma at presentation in the most affected eye

		Mild/Moderate		Advanced/End stage		Total	
		N	%	N	%	N	%
Mode of presentation	Symptomatic	15	16	77	84	92	100
	Referred	7	39	11	61	18	100
	Opportunistic	6	60	4	40	10	100
	First degree relative	3	27	8	73	11	100
Gender	Male	19	23	62	76	81	100
	Female	12	24	38	76	50	100
Age	50 years and above	10	13	70	87	80	100
	Less than 50 years	21	41	30	59	51	100
Ethnic group	Hausa	12	20	48	80	60	100
	Other	19	26	52	73	71	100
Occupation	Professional/civil servant/student/soldier/other	16	31	35	69	51	100
	Traders/Artisans/farmers	12	24	37	76	49	100
	Housewife/unemployed	3	10	28	90	31	100
Literacy	Not literate	10	13	65	87	75	100
	Literate	21	37	35	63	56	100
Family history of glaucoma	Yes	10	34	19	66	29	100
	No	21	21	81	79	102	100
Residence	Urban	27	28	70	72	97	100
	Rural	4	12	30	88	34	100
Place of residence	Within 10 kms of hospital	25	30	56	70	81	100
	10kms or more	6	12	44	88	50	100
Awareness of having glaucoma	No	19	23	63	76	82	100
	Yes	12	24	37	75	49	100
Knowledge about glaucoma	Good	11	50	11	50	22	100
	Poor	20	18	89	82	109	100
	Total	31	24	100	76	131	100

### Mode of presentation

Most participants attended because of symptoms (n = 92, 70 %), 18 (15 %) were formally referred with a diagnosis of glaucoma, 10 (8 %) were identified by the optometrists as they had a VCDR of ≥0.6, and 11 (8 %) were first-degree relatives of individuals with POAG who attended for assessment. Fifteen of the 18 referrals (14 % overall) were already receiving treatment as were 43/92 (33 % overall) presenting with symptoms. Sixty one participants therefore had a previous diagnosis of glaucoma, and 70 where newly diagnosed.

### Clinical findings at presentation

**Fig. 1:**
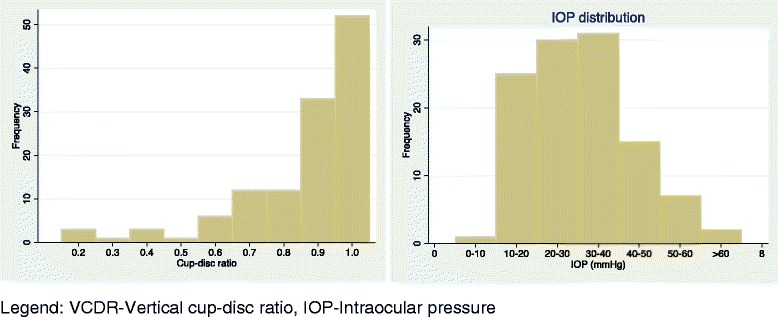
Distribution of VCDR and IOP in participants with primary open angle glaucoma, by eye. Legend: VCDR-Vertical cup-disc ratio, IOP-Intraocular pressure

Visual field testing was not possible in 73 (56 %) participants on account of loss of fixation or poor comprehension or manual dexterity. Intraocular pressures in all eyes with POAG ranged from 10 – 68mmHg (mean 31.9 +/- SD 12.4). The mean IOP of eyes already on treatment was lower than those not being treated (27.3mmHg, range 10-55mmHg versus 32.1mmHg, range 12–68mmHg). Mean VCDR in all eyes with POAG was 0.8 and 44 % of eyes had a VCDR of 1.0 (Fig. 1). 99 eyes (47 %) had a presenting VA of light perception or no light perception. 46 individuals (35 %) were blind in their better seeing eye (presenting VA <3/60). Overall 100 (76 %) participants were blind from glaucoma using WHO criteria i.e. had end stage or advanced glaucoma.

Forty-seven participants (36 %) had a positive history of blindness in their family, 29 of whom (22 %) gave a definite family history of glaucoma. The majority of patients (83 %) had poor awareness about glaucoma and how it is treated.

### Risk factors for advanced or end stage disease

In univariate analysis the following were associated with advanced or end stage disease in the worse eye: age >50 years, living >10 km from the hospital, some awareness of glaucoma, not being literate, being unemployed or a housewife and presenting with symptoms (Table 3). In multivariable analysis age and poor knowledge of glaucoma were independent risk factors for late presentation. Participants living more than 10km from the hospital were 2.94 times more likely have advanced/end-stage disease but this did not reach statistical significance (OR 2.94, 95 % confidence interval (CI) 0.93-9.32, p = 0.067). Living in urban areas (OR 0.35 95 % CI 0.11-1.07, p = 0.067) was protective in univariate analysis but this did not reach statistical significance. Findings were similar for least affected eyes (data not shown).

**Table 3:** Univariate and multivariable analysis of factors associated with advanced or end stage glaucoma at presentation, using the most affected eye

		Univariate analysis			Multivariable analysis		
		OR	95 % CI	P value	OR	95 % CI	P value
Sex	Male	1.0			1.0		
	Female	0.97	0.42-2.22	0.942	0.85	0.25-2.92	0.795
Age	Less than 50 years	1.0			1.0		
	>50 years	4.9	2.06-11.65	<0.001	3.45	1.24-9.58	0.017
Family history of glaucoma	Positive	1.0			1.0		
	Negative	0.53	0.21-1.29	0.16	0.39	0.12-1.28	0.122
Distance to hospital	10 km or less	1.0			1.0		
	More than 10km	3.27	1.24-8.67	0.017	2.74	0.75-10.09	0.129
Knowledge about glaucoma	Poor	1.0			1.0		
	Good	0.22	0.08-0.59	0.002	0.27	0.80-0.92	0.036
Literacy	Literate	1.0			1.0		
	Not literate	3.9	1.65-9.19	0.002	2.19	0.69-6.92	0.180
Awareness of having glaucoma	Yes	1.0					
	No	1.07	0.46-2.46	0.864			NS
Ethnicity	Hausa	1.0					
	Other	0.80	0.52-1.24	0.327			NS
Occupation	Professional/civil servants etc.	1.0					
	Traders/Artisans/farmers	1.40	0.58-3.39	0.301			NS
	Housewife/unemployed	4.27	1.12-16.12	0.032			NS
Mode of presentation	Referred	1.0					
	Symptomatic	3.26	1.09-9.78	0.034			NS
	Opportunistic	0.42	0.87-2.06	0.288			NS
	First degree relative	1.69	0.33-8.67	0.525			NS
Residence	Rural	1.0					
	Urban	0.35	0.11-1.07	0.067	0.65	0.15-2.79	0.56

*OR* = odds ratio; 95 % *CI* = 95 % confidence interval; *NS* = not statistically significant

### Acceptance of surgery and adherence to medical treatment and follow up

Forty six participants were offered treatment for pain control for end stage disease and 85 were offered treatment to preserve visual function. Among the latter, 14 were offered topical treatment and 71 were offered trabeculectomy as the treatment of choice. Only five (8 %) agreed to surgery. Reasons for not accepting surgery included fear (37 %), wanting to continue current treatment (27 %), cost (15 %), no time (6 %), being too old (4 %) or needed to consult their family (4 %). Only one patient returned for trabeculectomy. Among the 85 participants started on topical medication, 32 (24 %) returned at one month. 72 % reported excellent compliance but only 56 % of glaucomatous eyes had an IOP of <21mmHg.

## Discussion

The majority of patients in this study presented to hospital because of symptoms, with many already blind in at least one eye, which confirms the findings of other studies in Africa. Our series of patients were far more likely to be blind at presentation than cases who present to eye departments were there are primary care providers such as optometrists, who can detect and refer individuals suspected as having glaucoma. For example, in a study from the UK, 91 % of eyes had a visual acuity of 6/12 or better at presentation [13].

In this study 38 % of patients were not aware they had glaucoma before the study and only 17 % had any knowledge about glaucoma, being similar to a study in Ethiopia, [14] and many population based studies in other parts of Africa and other regions of the world, including industrialized countries [15–17]. The finding that some patients already being treated for glaucoma had poor knowledge of the condition reflects poor counselling, which is a challenge in northern Nigeria as there is not a local word in the Hausa language for glaucoma. Lack of knowledge of glaucoma is greater amongst people with low socioeconomic status [18] who need to be targeted for interventions, as greater knowledge of glaucoma has been associated with greater adherence to treatment, in Oman for example [19]. Indeed, poor knowledge has also been reported among eye health workers [20].

In this study older age and poor knowledge of glaucoma were independent risk factors for late presentation in multivariable analysis. Living more than 10km from the hospital increased the risk in univariate analysis, as did not being literate, presenting with symptoms and being unemployed. These associations did not reach statistical significance in multivariable analysis, possibly reflecting the relatively small sample size. Other studies in Africa have shown a relationship between late presentation of POAG with low levels of education and low socioeconomic status, [21] but these factors were not independent risk factors in our study. Greater awareness needs to be created about glaucoma in the population to promote earlier presentation.

Acceptance of surgical treatment for glaucoma is a problem in Africa, [5, 6, 22] as was confirmed in this study. In Tanzania, individuals identified with glaucoma during a population-based survey were referred for trabeculectomy. Acceptance of trabeculectomy was 46 % lower than cataract surgery (80 %) but nevertheless high for glaucoma in Africa. Many patients in Africa do not understand why they are offered surgery in the better eye, particularly if they are already blind in the other eye. In addition, the Hausa word for surgery, which translates as butchering, has very negative connotations. Acceptance of eye surgery is also poor for other eye conditions e.g., for trichiasis surgery [23] and rumours can play a role in patient’s decision making [24].

A trial in India demonstrated that acceptance of treatment increased with education [25]. Implications are that counselling or heath education, which dispels rumours and overcomes fear can improve acceptance of treatment. Counselling techniques such as Motivational Interviewing, shown to be promising in a range of conditions [26–28], could play a role. The aim of Motivational Interviewing is to explore and resolve patient’s ambivalence and promote his or her own motivation for change. This hypothesis is currently being explored in a randomised trial of surgical interventions for glaucoma in ATBUTH, Bauchi [29].

Adherence is defined by WHO as “the extent to which a person’s behaviour corresponds with agreed recommendations from a health care provider” [30]. Self-reported adherence in this study was relatively high at 72 %, but as this did not correspond with IOP reduction this finding is questionable. Adherence to treatment is a global problem. Indeed, a National Health Service (United Kingdom) report estimated that 30-50 % of prescribed medication is not taken as recommended [31], and a study in the United States of America showed that nearly half of patients on ocular hypotensive therapy discontinued within six months [32]. Non-compliance with glaucoma treatment is common but more common in patients of African descent [33]. Motivational interviewing may also have a role to play in increasing adherence to topical medication for glaucoma in Africa and other settings.

In our study only a quarter of patients attended follow up at one month, which was lower than reported in Ibadan, Nigeria [34]. In our study reasons for non-attendance were not investigated but in the Ibadan study reasons included lack of transport, fear of surgery, no improvement with treatment, feeling better or no improvement with treatment. Similar findings have been reported from India [35]. In the Ibadan study default was more likely among younger patients, males and those from long distances while those with severe disease were more likely to attend. Poor follow up after surgery has also been reported in Tanzania, for example [22]. Poor knowledge has also been linked to poor follow up among glaucoma patients [36].

The implications of our study are that counselling is needed at the time of diagnosis so that patients understand the purpose and importance of follow up. Our study supports the recommendation of others of the need for a once off treatment for glaucoma in Africa that does not require regular follow up [5, 37].

This study demonstrates some of the problems encountered in managing glaucoma in Africa. Strategies which promote earlier detection, such as opportunistic screening or examination of first degree relatives, coupled with counselling, may promote greater acceptance and adherence to treatment. Public awareness needs to be increased while services and expertise are being developed, which needs to include a once-off, relatively non-invasive treatment of proven effectiveness which is acceptable to patients, such as laser trabeculoplasty or transscleral diode laser cyclophotoablation.

In Africa, until glaucoma care becomes a sub-speciality supported by technology for assessment, grading will need to rely primarily on estimation of VCDR and VA, supported by VF assessment when possible, with the goal of avoiding bilateral blindness. This is supported by our study where only 36 % of patients were able to perform VF testing well enough to allow glaucoma to be diagnosed based on characteristic VFDs.

Strengths of this study are that it was a single centre, prospective study, and one ophthalmologist undertook all diagnostic examinations. Limitations include the relatively small sample size and the diagnosis of glaucoma was based mainly on VCDR and IOP as VF testing was not possible in many participants.

## Conclusion

Majority of glaucoma patients in Africa only report to hospital when they have symptoms of loss of vision. This late presentation coupled with poor adherence to medical treatment and acceptance of surgery means that many continue to go blind despite reporting to hospital. To prevent glaucoma blindness strategies are required which promote earlier detection, with counselling to promote acceptance of and adherence to treatment.

## Abbreviations

ATBUTH: Abubakar Tafawa Balewa University Teaching Hospital
CI: Confidence Interval
IOP: Intraocular Pressure
Km: Kilometre
NS: Not significant
OR: Odds ratio
POAG: Primary Open Angle Glaucoma
SD: Standard deviation
VA: Visual acuity
VF: Visual field
VFA: Visual field analysis
VFD: Visual field defect
VCDR: Vertical cup-disc ratio
WHO: World Health Organisation
